# Soil Organic Carbon Increases in Semi-Arid Regions while it Decreases in Humid Regions Due to Woody-Plant Encroachment of Grasslands in South Africa

**DOI:** 10.1038/s41598-018-33701-7

**Published:** 2018-10-19

**Authors:** Admore Mureva, David Ward, Tiffany Pillay, Pauline Chivenge, Michael Cramer

**Affiliations:** 1 School of Life Sciences, University of KwaZulu-Natal, P. Bag X01, Scottsville, 3209 South Africa; 2 Present Address: Bindura University of Science Education, P. Bag 1020, Bindura, Zimbabwe; 3 Biological Sciences Department, Kent State University, Kent, 44242 USA; 4 Present Address: International Crops Research Institute for the Semi-Arid Tropics, P. O. Box 776, Bulawayo, Zimbabwe; 5 Present Address: Department of Biological Sciences, University of Cape Town, Cape Town, 7700 South Africa

## Abstract

Grasslands and savannas are experiencing intensive land-cover change due to woody plant encroachment. This change in land cover is thought to alter soil carbon (C) and nitrogen (N) storage in these ecosystems. Some studies have reported a negative correlation between soil C and N and mean annual precipitation while others have indicated that there is no relationship with mean annual precipitation. We quantified the changes in C and N pools and δ^13^C and δ^15^N values to a depth of 1 m in pairs of encroached and adjacent open grassland sites along a precipitation gradient from 300 mm to 1500 mm per annum in South Africa. Our study showed a negative correlation between changes in soil organic C stocks in the 0–100 cm soil layer and mean annual precipitation (MAP). The most humid site (1500 mm MAP) had less C in shrub-encroached sites while the drier sites (300–350 mm MAP) had more C than their paired open grasslands. This study generally showed soil organic C gains in low precipitation areas, with a threshold value between 750 mm and 900 mm. Our threshold value was higher than that found in North America, suggesting that one cannot extrapolate across continents.

## Introduction

Soils are a major reservoir of terrestrial carbon (C), storing more C (2 344 Gt C, up to 3 m depth) than terrestrial biomass (560 Gt C) and atmospheric pools (750 Gt C)^1–3^. A small change in soil organic C (SOC) may have a great impact on atmospheric C and subsequently on the earth’s climate^4^. Land-cover change is one of the main factors that can alter SOC content^5,6^. One of the major sources of land-cover change, especially in tropical and subtropical regions, is encroachment by native woody plant species. In South Africa, this problem is particularly acute; encroachment affects about 10–20 million ha, seriously reducing the productivity for a country where >70% of its agricultural area is largely grazing lands^7^.

Jackson *et al*.^8^ examined the effects of vegetation change on SOC at six paired grassland and invaded woody sites along a precipitation gradient in the south-western USA, testing relationships between biomass C gains and SOC losses for common native woody invaders, such as *Prosopis* (mesquite), *Larrea* (creosote) and *Juniperus* (juniper) species. In that study, they found that there was a greater increase in SOC stored in the encroached sites than in the open grasslands in drier locations (i.e., below ~400 mm mean annual precipitation (MAP)). However, Jackson *et al*.^8^ also observed lower values of SOC in encroached sites than in open grasslands in the more humid locations. Other data show that generally organic C and total N pools in soils beneath shrub canopies increase linearly with time since shrub establishment^9^. Findings from several studies^10,11^ indicated that woody plant encroachment is a potentially, but highly uncertain, carbon sink. A subsequent meta-analysis of SOC data on woody plant encroachment found that the change in soil organic C in response to woody plant encroachment was highly variable and unrelated to MAP^12^.

Soil bulk density and clay content mediate the magnitude and direction of change in SOC with woody encroachment. In encroached grasslands SOC decreased as soil bulk density increased^12^ while SOC accumulated with increasing clay content^13,14^. Barger *et al*.^12^ reported that SOC accumulated linearly with increasing clay content (r^2^ = 0.76), presumably reflecting greater occlusion and protection of organic matter afforded by clay micelles^13,14^. They also found that soil C accumulation decreased as soil bulk density increased, with C gains (r^2^ = 0.52) being confined to soil densities less than 1.6 g cm^−3^.

Woody plant encroachment in tropical grasslands usually alters the ecosystem from C_4_ plants (African grasses use this photosynthetic pathway) to C_3_-dominated woody vegetation^13,14^. The change in the dominant photosynthetic pathway offers a unique opportunity to use the natural abundance of ^13^C to evaluate changes in C cycles due to woody plant encroachment of grasslands. Variations in ^13^C/^12^C ratios of plants utilising the C_3_ and C_4_ photosynthetic pathways provides a natural tracer when a C_4_ community type (δ^13^C ≈ −14‰) is replaced by a C_3_ community (δ^13^C ≈ −27‰) or *vice versa*^15,16^, allowing for the differentiation of the C derived from C_4_ vegetation from C derived from C_3_^8,17,18^.

SOC in encroached grasslands can also be enhanced by an increase in available soil nitrogen. Change in nitrogen availability in ecosystems is likely to drive net primary productivity and thus C sequestration^19^. The frequent increase of N_2_-fixing woody species in grasslands^20,21^ has strong potential for altering the N-cycle, primary production, and other key ecosystem processes^21,22^. Plant species are likely to influence ecosystem N cycling via alterations in N-use efficiency and by changing N inputs and losses^23^. Changes to the soil total N pool via the shift in the balance of N inputs versus losses should be apparent in the natural abundance of ^15^N in the plant-soil system^21^.

In this study, we quantified and compared C and N pools (measured as concentrations and mass stocks) between adjacent woody- encroached and open-grassland plots along a precipitation gradient (300 mm to 1500 mm per annum) in South Africa. The major encroaching species in our sites (except at the 300 mm MAP site in Middelburg) were potentially N_2_-fixing shrubs (*Vachellia karroo*, *Senegalia mellifera*, *V*. *tortilis* and *V*. *erioloba*). We focused on responses in C sequestration, and in particular its relationship with MAP, as a key continental scale driver of ecosystem function^24^. We recorded SOC, N, δ^13^C and δ^15^N at depth intervals of 0–10, 10–30, 30–60 and 60–100 cm and determined the δ^13^C and δ^15^N isotopic ratio in plant leaves and plant litter. We also determined the contribution of C_4_ plants to SOC in the encroached grasslands. Our study sites contained mostly warm-season C_4_ grasses (Table 1). The sites in decreasing order of precipitation were KwaMbonambi (1500 mm yr^−1^), Stanger (900 mm yr^−1^), Bergville (750 mm yr^−1^), Bloemfontein (500 mm yr^−1^), Pniel (350 mm yr^−1^) and Middelburg (300 mm yr^−1^). At each site, we examined six encroached and six open grasslands. We predicted that in encroached grasslands there would be a negative correlation between changes in SOC and MAP^8^. We also predicted a negative correlation between C change due to woody plant encroachment and soil bulk density and a positive correlation between C change due to woody encroachment and silt and clay content^10^. We predicted similar relationships for soil N because of the tight relationship between soil C and N^8^. We also predicted that δ^13^C will become less enriched in shrub-encroached grasslands compared to open grasslands because of transition from C_4_ grasses to C_3_ woody-shrubs and trees^15,16^.

**Table 1:** Study site descriptions.

Site	GPS coordinates	Annual Rainfall (mm)	Temperature (°C)		Biome	Soil Characteristics	Major plant species	General land management
			Min	Max				
Kwambonambi	28°49′61″S 32°16′97″E	1500	3.5	35	Maputaland wooded grasslands	Quaternary redistributed sands supporting yellowish redistributed sands of the Berea formation	*Sporobolus fimbriatus*, *Digitaria natalensis *(grass species); *Diospyros lycioides* (shrub)*; Terminalia sericea* (tree)	*Protected area*, *privately managed*, *generally excluded from fires*, *herbivory activities from wild and livestock*
Stanger	29°18′59″S 31°22′13″E	900	5.8	32.6	KwaZulu-Natal Coastal Belt	Ordovician Natal group sandstone	*Themeda triandra*, *Aristida junciformis* (grass); *Vachellia karroo* (*tree*)	*Protected area*, *privately managed*, *generally excluded from fires*, *herbivory from wild animals and livestock*
Bergville	28°79′06″S 29°38′98″E	750	5.8	32.6	KwaZulu-Natal moist grasslands	Ordovician Natal group sandstone	*Themeda triandra*, *Hyparrhenia hirta* (grasses)*; Vachellia karroo*, *Vachellia sieberiana* (*trees*)	*Protected area*, *generally excluded from fire*, *herbivory from wild animals and livestock*
Bloemfontein	28°59′17″S 26°16′54″E	450	0	32	Bloemfontein dry grasslands	Sedimentary mudstones and layers of sandstone	*Aristida congesta*, *A*. *diffusa*, *Cynodon dactylon* (grass)*; Vachellia karroo* (*tree*)	*Protected area*, *privately managed*, *generally excluded from fire*, *herbivory from wild animals and livestock*
Pniel	28°34′50″S 24°30′30″E	350	−4.1	37.5	Kimberley thornveld	Sandy-loam soils of the Hutton soil form	*Eragrotis curvula*, *Schmidtia pappophoroides* (grass)*; Vachellia erioloba*, *Vachellia tortilis*, *Vachellia karroo*, *Senegalia mellifera*, *Tarchonanthus camphoratus* (trees)	*Communal land*, *very low frequency of fireherbivory from livestock*
Middelburg	31°25′98″S 24°58′82″E	300	−7.2	36.1	Eastern Upper Karoo	Sandy to loam soils of the Hutton soil form	*Aristida* and *Eragrostis* (grass); *Searsia erosa*, *S*. *burchellii*, *Diospyros lycioides* and *Eriocephalus ericoides* (shrubs)	*Protected area*, *privately managed*, *generally excluded from fire*, *herbivory from wild animals and livestock*

**Figure 1:**
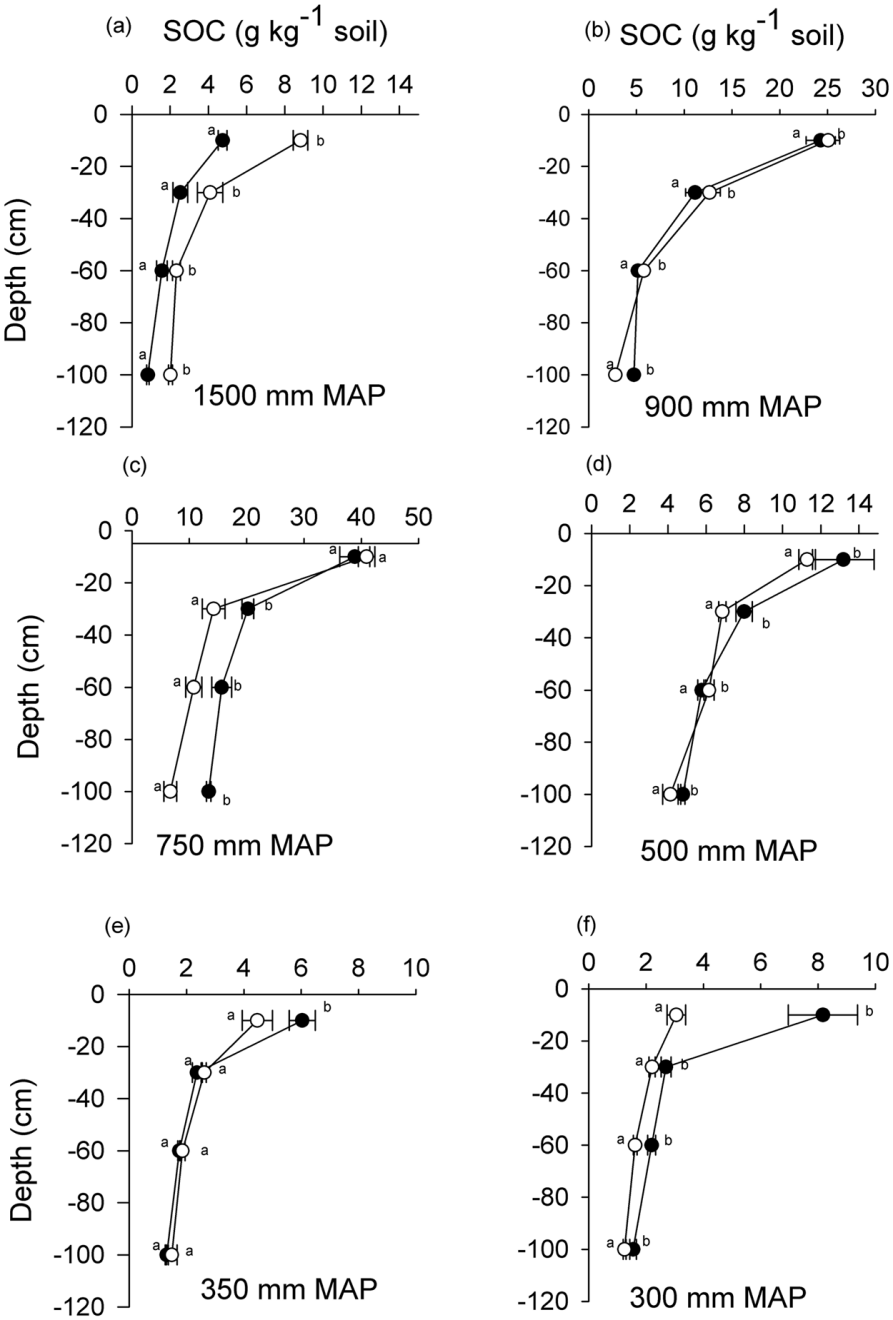
Soil organic carbon concentrations (g C kg^**−**1^ soil) vs. depth (cm) along a precipitation gradient (300–1500 mm MAP) in encroached grasslands (black circles) and open grasslands (open circles). Error bars represent standard errors. Some encroached and open grassland data points at the 350 and 300 mm sites completely overlap. Means within the same depth different letters are significantly different (Tukey *post hoc* test, p < 0.05).

At the most humid site (1500 mm MAP), in the 0–10 cm layer, SOC concentration and SOC stocks were significantly greater (F_1, 10_ = 83.70, P < 0.0001 and F_1, 10_ = 103.9, P < 0.0001) in the open grasslands than in shrub-encroached grasslands (Fig. 1). This humid site lost 50.5% of SOC stocks in the 0–10 cm soil layer (Table 2). The loss in SOC stocks to 100 cm depth due to woody plant encroachment at this humid site was almost 50%. At the 900, 750 and 500 mm MAP sites there were generally no differences in SOC concentrations and stocks between encroached and open grasslands in the uppermost soil layer (Fig. 1, Table 2). However, the 750 and 500 mm MAP sites gained carbon stocks slightly at 0–1 m depth, ranging between 9.2% and 25%. In the semi-arid sites of 350 and 300 mm MAP, SOC concentration in the 0–10 cm soil layer was higher (F_1,10_ = 5.06, P = 0.048 and F_1,10_ = 16.73, P = 0.002, respectively) in encroached grassland than in open grasslands (Fig. 1). The 350 and 300 mm MAP sites had higher SOC stocks (F_1, 10_ = 11.26, P < 0.007 and F_1, 10_ = 11.26, P < 0.022) in the encroached grasslands compared to open grasslands in the upper soil layer.

**Table 2:** Soil organic C and N stocks (Mg/ha) to 1 m depth and % change in C and N after shrub encroachment.

MAP (mm)	0–10 cm			10–30 cm			30–60 cm			60–100			0–100 cm		
	Shrub	Grass	%change	Shrub	Grass	%change	Shrub	Grass	%change	Shrub	Grass	% change	Shrub	Grass	% change
**Soil organic carbon stocks**															
1500	4.9 ± 0.24^a^	9.9 ± 0.4^b^	−50.5	6.0 ± 0.9^a^	10.5 ± 1.7^b^	−43.5	5.9 ± 1.0^a^	9.8 ± 0.8^b^	−40.4	3.5 ± 0.3^a^	9.9 ± 0.5^b^	−64.4	20.3 ± 2.0^a^	40.2 ± 2.2^b^	−49.6
900	27.6 ± 1.7^a^	30.5 ± 1.5^b^	−9.5	25.3 ± 2.3^a^	33.6 ± 3.0^b^	−24.7	17.1 ± 0.9^a^	23.3 ± 2.30	−26.4	21.6 ± 0.9^a^	13.5 ± 2.5^b^	59.9	91.6 ± 2.7^a^	100.9 ± 4.0^b^	−9.2
750	40.0 ± 2.7^a^	40.9 ± 1.7^a^	−18.0	42.1 ± 2.3^a^	35.2 ± 4.9^b^	19.5	55.8 ± 6.2^a^	38.7 ± 5.0^b^	44.3	62.1 ± 1.7^a^	36.5 ± 6.1^b^	70.4	200.1 ± 3.6^a^	159.1 ± 3.5^b^	25.7
500	16.0 ± 2.0^a^	12.8 ± 0.5^a^	24.8	21.2 ± 1.1^a^	16.0 ± 0.9^b^	32.4	19.1 ± 0.7^a^	24.6 ± 1.1^b^	−22.4	23.1 ± 0.5^a^	19.0 ± 1.8^b^	21.7	79.4 ± 3.2^a^	72.4 ± 3.1^b^	9.7
350	7.8 ± 0.59^a^	5.0 ± 0.59^b^	55.9	6.1 ± 0.40^a^	6.0 ± 0.13^a^	2.2	6.7 ± 0.21^a^	6.6 ± 0.34^a^	1.7	6.9 ± 0.30^a^	7.0 ± 0.85^a^	−0.5	27.5 ± 1.62^a^	24.5 ± 1.76^b^	12.3
300	8.2 ± 1.2^a^	3.9 ± 0.4^b^	108.9	6.4 ± 0.4	6.1 ± 0.3^b^	5.2	8.2 ± 0.5^a^	6.0 ± 0.4^b^	37.2	8.0 ± 0.6^a^	7.4 ± 0.3^a^	9.3	30.92.2^a^ ±	23.4 ± 1.7^a^	32.1
**Total nitrogen stocks**															
1500	0.5 ± 0.2^a^	0.6 ± 0.4^b^	−27.2	0.6 ± 0.5^a^	0.7 ± 0.01^b^	−23.9	0.6 ± 0.5^a^	0.8 ± 0.2^b^	−23.2	0.4 ± 0.1^a^	0.8 ± 0.4^b^	−53.5	2.0 ± 0.6^a^	3.0 ± 0.7^b^	−32.6
900	4.2 ± 1.0^a^	2.3 ± 0.6^b^	81.0	2.4 ± 0.5^a^	3.1 ± 0.7^b^	−20.3	2.6 ± 0.5^a^	2.8 ± 0.7^a^	−7.1	3.6 ± 0.8^a^	3.1 ± 0.8^a^	16.3	12.8 ± 1.1^a^	11.3 ± 1.07^b^	13.6
750	3.7 ± 0.4^a^	4.1 ± 0.8^a^	−10.9	3.3 ± 0.7^a^	3.1 ± 0.8^a^	7.1	4.5 ± 0.9^a^	3.8 ± 1.1^b^	19.6	4.6 ± 0.5^a^	3.8 ± 0.9^b^	19.9	16.1 ± 1.1^a^	14.8 ± 1.0^b^	8.6
500	1.4 ± 0.4^a^	1.3 ± 0.3^a^	8.4	1.7 ± 0.6^a^	2.1 ± 0.5^b^	−17.1	2.0 ± 0.4^a^	2.5 ± 0.3^b^	−21.2	2.1 ± 0.5^a^	2.6 ± 0.4^b^	−19.0	7.2 ± 0.9^a^	8.5 ± 0.8^b^	−14.9
350	0.5 ± 0.1^a^	0.4 ± 0.0^b^	33.0	0.6 ± 0.2^a^	0.6 ± 0.1^a^	4.1	0.8 ± 0.1^a^	0.8 ± 2^a^	−2.5	0.9 ± 0.3^a^	0.8 ± 0.1^a^	6.0	2.7 ± 0.1^a^	2.6 ± 0.1^b^	6.7
300	1.1 ± 0.1^a^	0.7 ± 0.1^b^	47.1	1.2 ± 0.2^a^	1.0 ± 0.3^a^	18.5	1.3 ± 0.1^a^	1.0 ± 0.1^b^	36.6	1.1 ± 0.1^a^	1.1 ± 0.1^a^	6.7	4.8 ± 0.2^a^	3.8±0.1^b^	25.2

**Figure 2:**
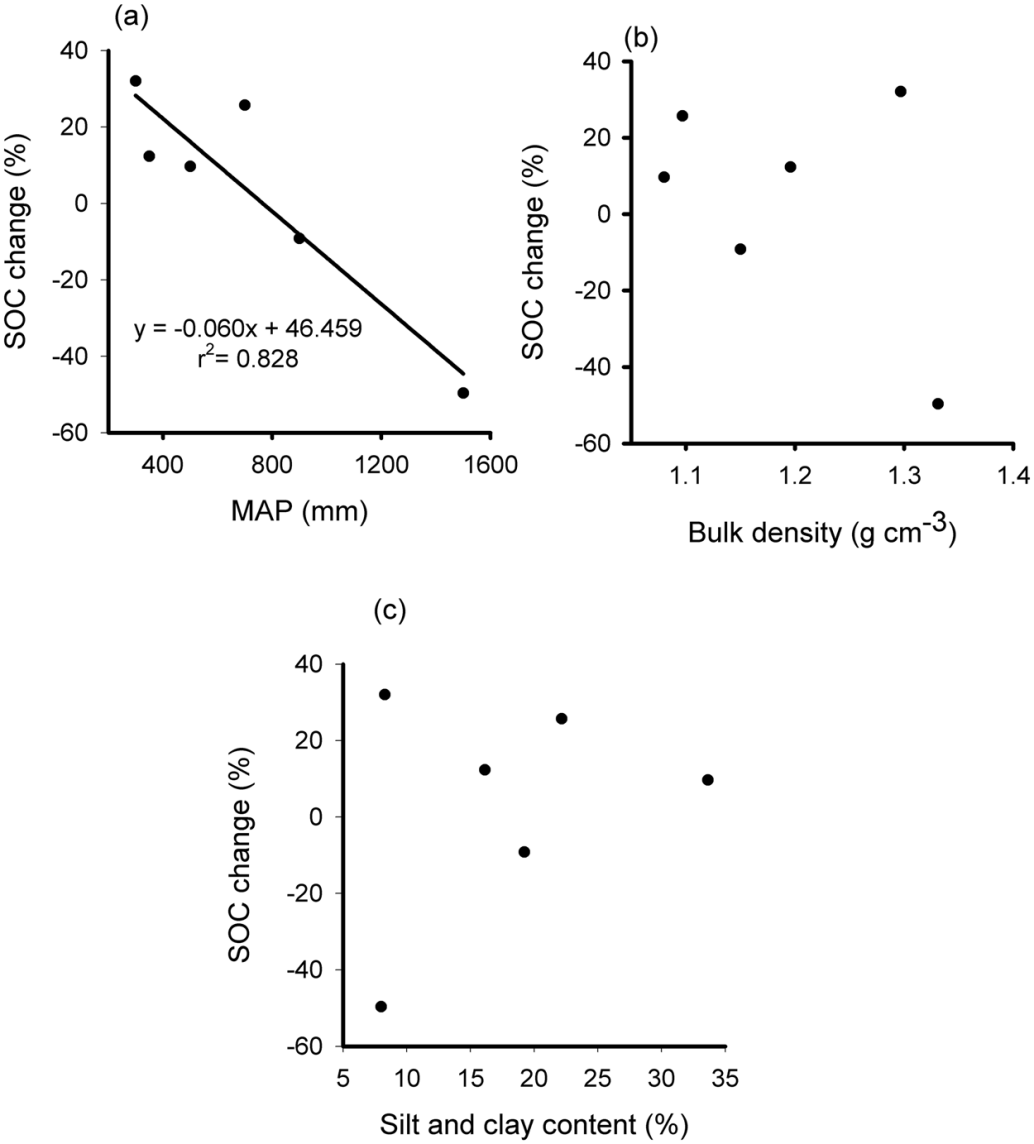
Relationship between changes in SOC stocks (%) at 0–100 cm depth with woody encroachment in pairs of encroached and open grasslands and (**a**) mean annual precipitation (MAP) in mm; (**b**) silt and clay content as percentage; (**c**) bulk density in g cm^**−**3^. A positive value is achieved because there is more SOC in encroached soils than in open grassland soils and *vice versa*.

There was a significant negative correlation between change in SOC stocks in open versus encroached grasslands in the 0–100 cm soil layer and mean annual precipitation (MAP) (r^2^ = 0.83, P = 0.012 (Fig. 2a)). In contrast, there was no significant relationship between the change in SOC stocks (between open and encroached grasslands) and soil bulk density (r^2^ = 0.05, P = 0.68 (Fig. 2b)) and between change SOC stocks and silt and clay content (r^2^ = 0.02, P = 0.77 (Fig. 2c)).

**Figure 3:**
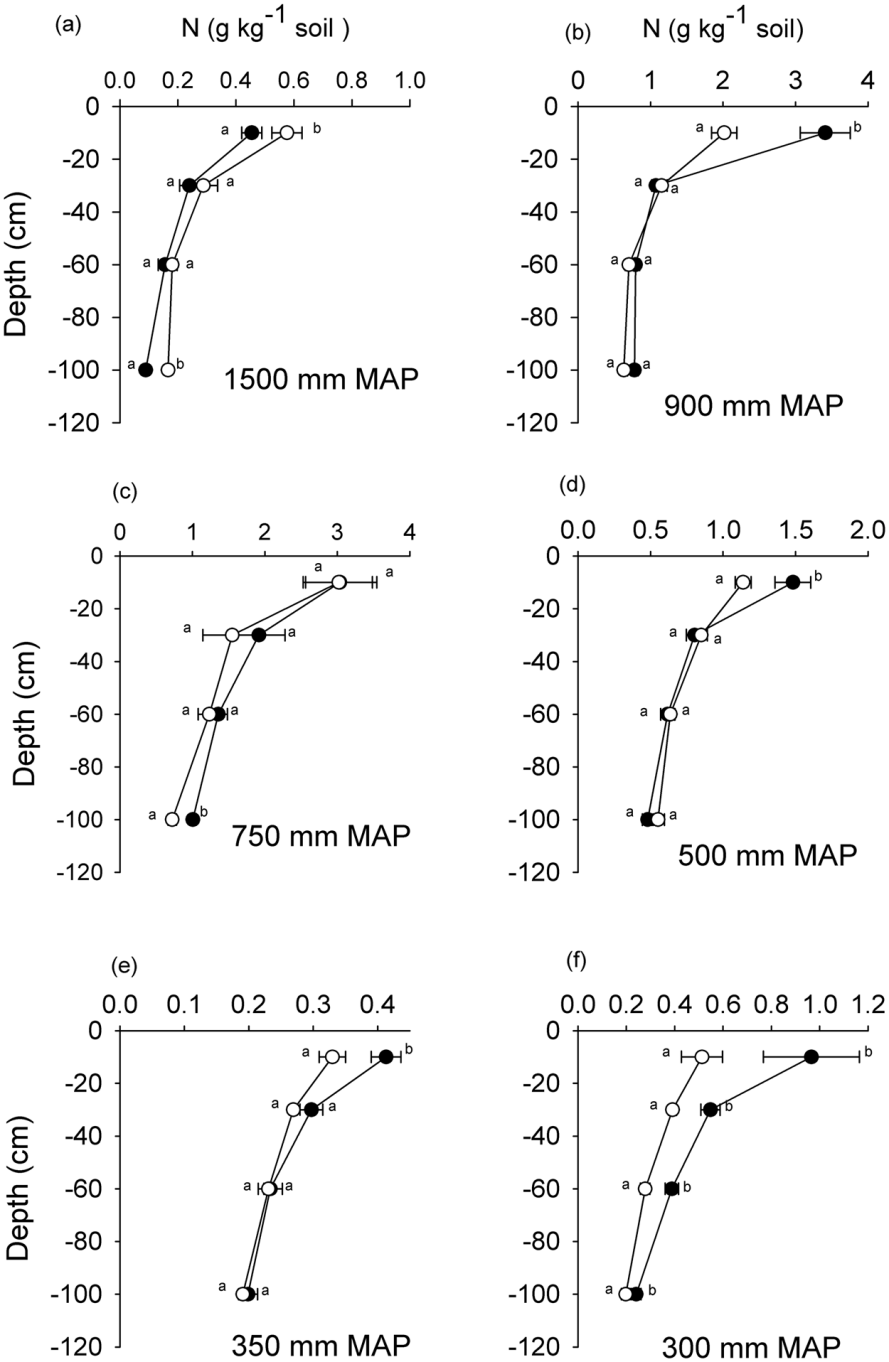
Soil total-N concentrations (g N kg^−1^ soil) with depth (cm) along a precipitation gradient (300–1500 mm MAP) in encroached grassland (black circles) and open grasslands (open circles). Means at the same depth with different letters are significantly different (Tukey *post hoc* test, p < 0.05).

Nitrogen was significantly higher in shrub-encroached grasslands than in the open grasslands in the 0–10 cm soil layer in the 900, 500, 350 and 300 mm MAP sites (Fig. 3). The correlation between the change in total N stocks of encroached versus open grasslands in the 0–100 cm soil layer and MAP tended to be negative and marginally significant (r^2^ = 0.62, P = 0.064; data not shown). There was no significant relationship between total N change between encroached and open grasslands and soil bulk density (r^2^ = 0.001, P = 0.89) or between total N change and silt and clay content (r^2^ = 0.026, P = 0.76).

**Figure 4:**
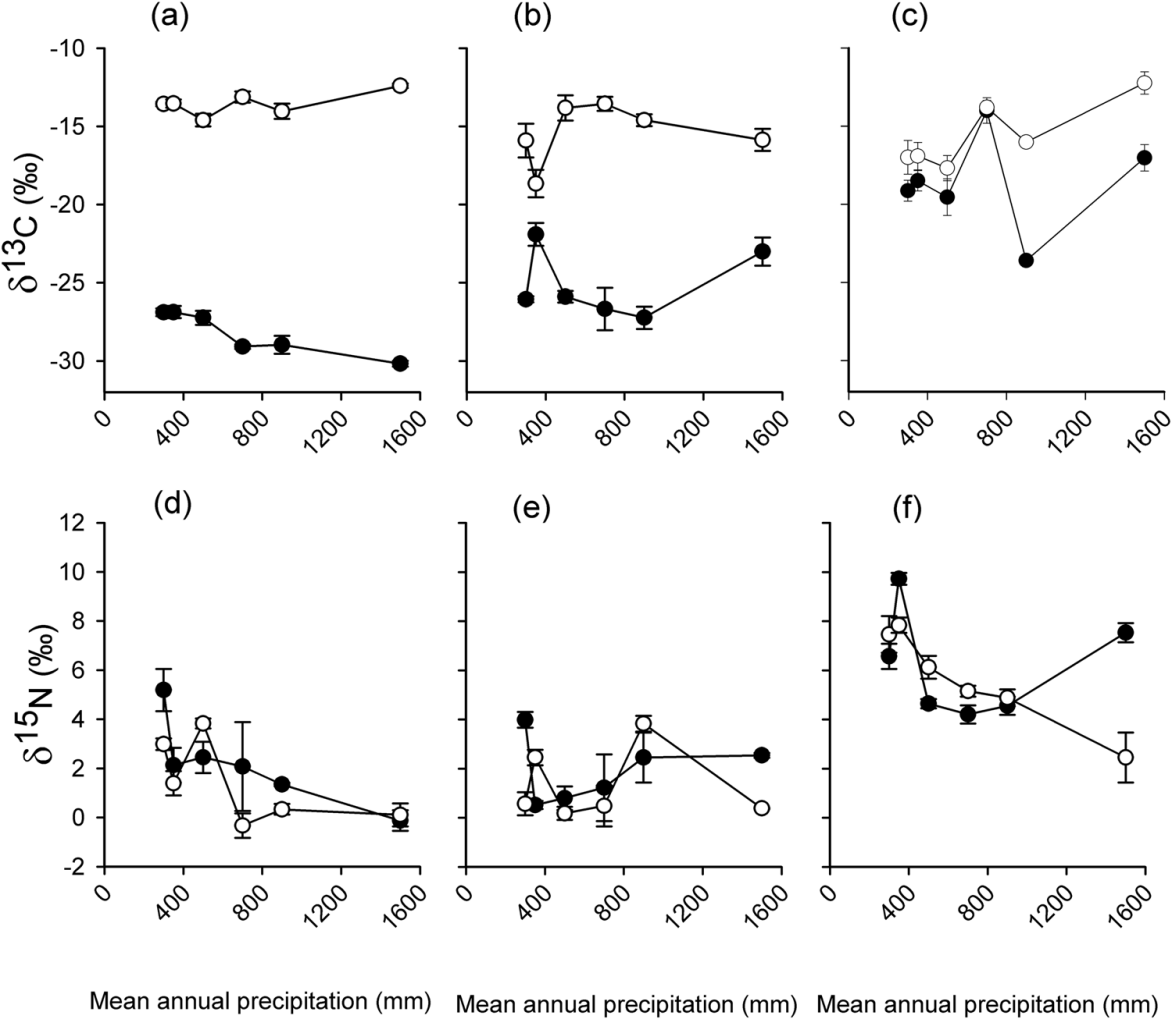
δ^13^C (‰) and δ^15^N (‰) in open grasslands (open circles) and encroached grasslands (black circles) in plant material (**a**,**d**), plant litter (**b**,**e**) and top soil (**c**,**f**).

In the encroached grasslands, δ^13^C was generally more negative in the plant foliage and plant litter compared to the top soil. The open grassland showed the opposite trend, with the plant material being more enriched in δ^13^C than the top soil (Fig. 4). δ^15^N was generally more enriched in the top soil compared to the plant material in both the encroached and open grasslands (Fig. 4).

**Figure 5:**
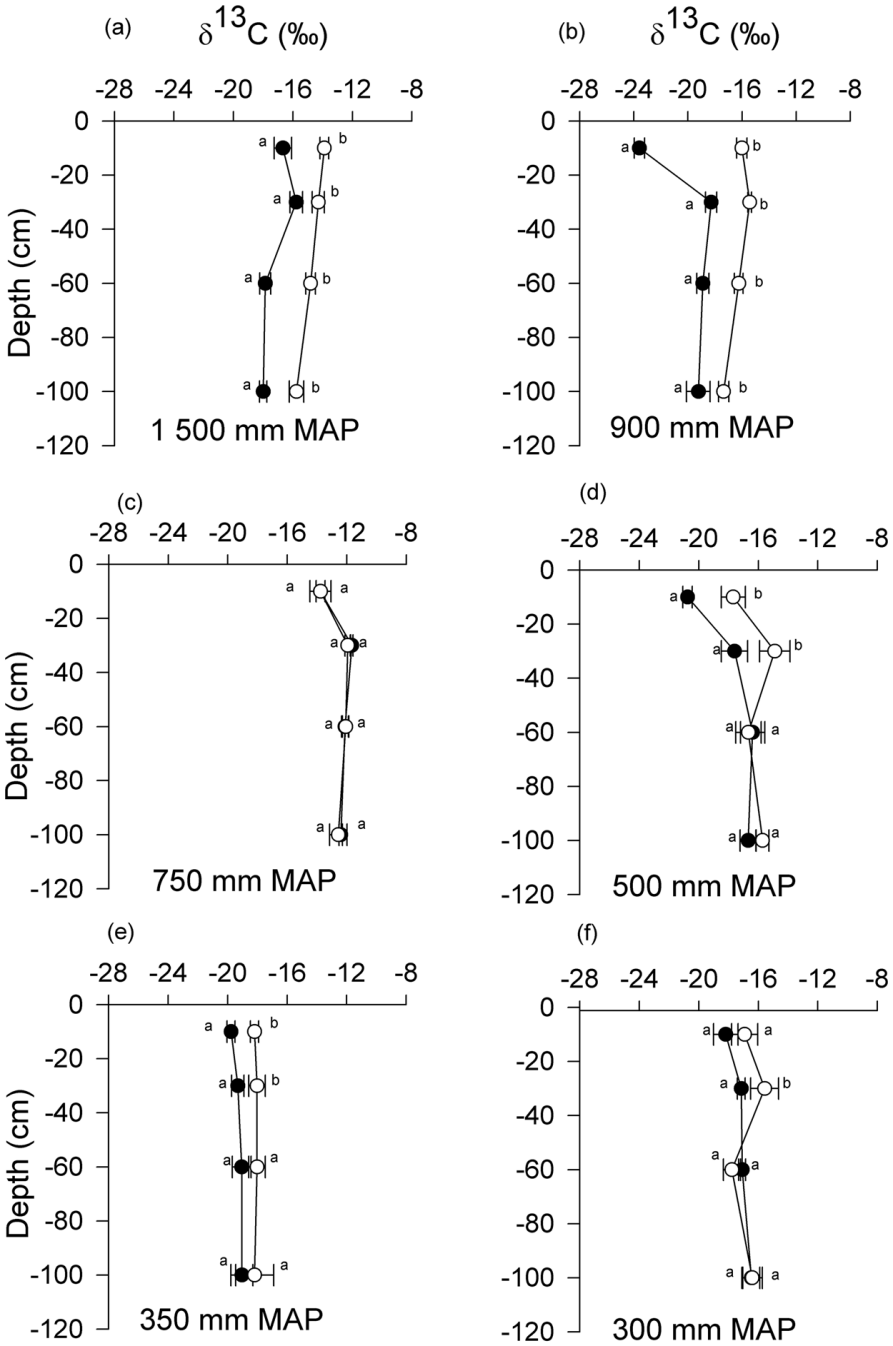
δ^13^C (‰) values in encroached grassland (black circles) and open grasslands (open circles) with depth (cm) in South Africa. Error bars are standard errors of the mean. The reference standard was Pee Dee Belemnite. Means at the same depth with different letters are significantly different (Tukey *post hoc* test, p < 0.05).

**Figure 6:**
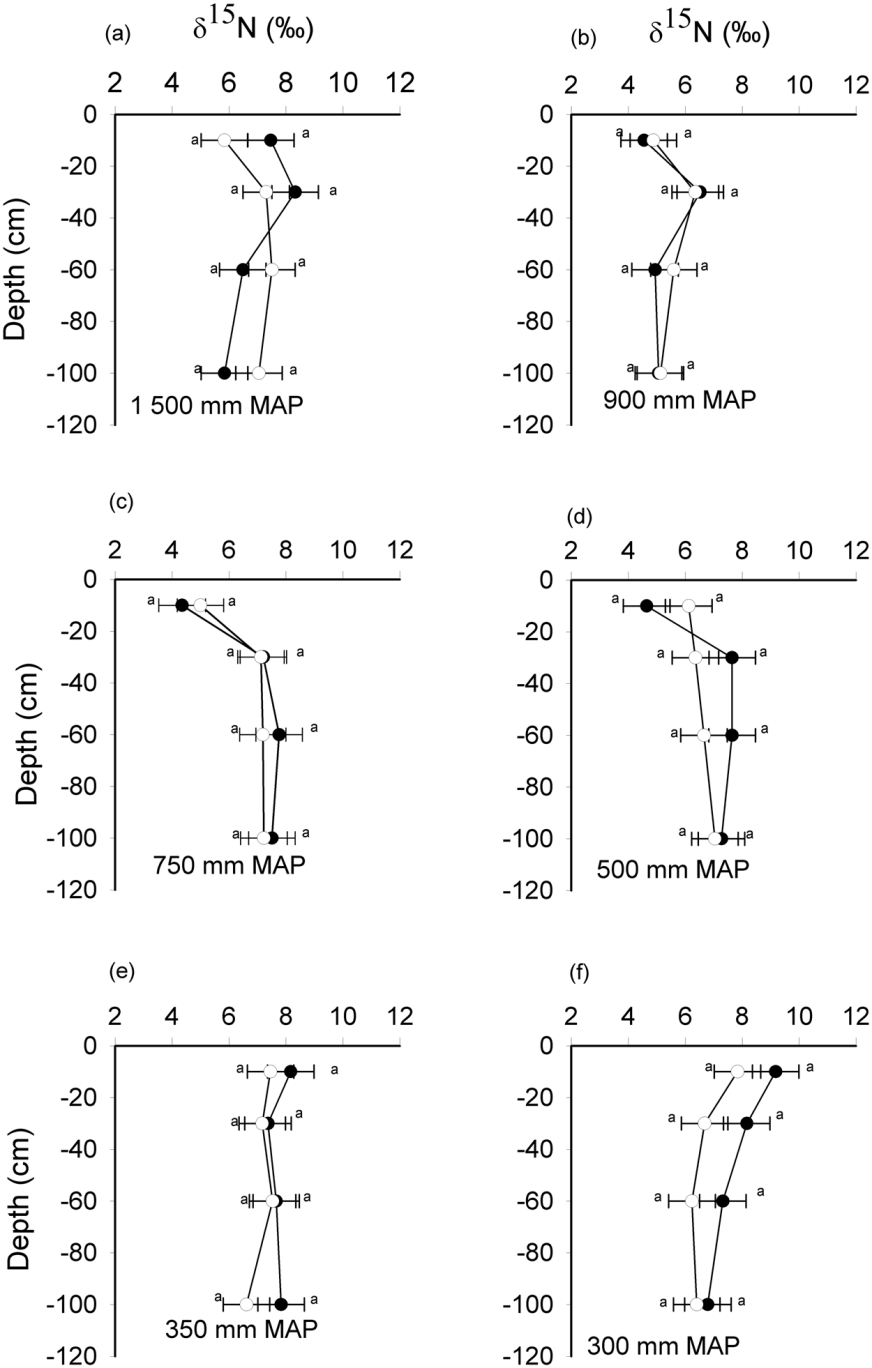
δ^15^N (‰) values in encroached grassland (black circles) and open grasslands (open circles) with depth (cm) in South Africa. Error bars are standard errors of the mean. The reference standard was atmospheric nitrogen. Means at the same depth with the same letter are not significantly different (Tukey *post hoc* test, p < 0.05).

At 0–10 cm depth, the δ^13^C values were significantly different between encroached and open grasslands in the 1500, 900, 500 and 350 mm MAP sites while in the 300 and 750 mm MAP sites there was no significant difference in δ^13^C between the two encroached and open grasslands (Fig. 5). There was generally δ^13^C enrichment with increasing depth across the precipitation gradient. There was no significant correlation between the change in δ^13^C between encroached and open grasslands and mean annual precipitation (MAP) (r^2^ = 0.03, P = 0.35). There was no significant enrichment of δ^15^N with increasing depth in both the encroached and open grasslands (Fig. 6) across all sites. Encroached grasslands had a significantly higher δ^15^N than open grasslands in the 500 MAP site (F_1, 10_ = 8.72, P = 0.01) in the 0–10 cm soil layer (Fig. 6). δ^15^N change between encroached and open grasslands was also not significantly correlated with MAP (r^2^ = 0.20, P = 0.46). Although there was a positive relationship between δ^15^N change and silt and clay content, the relationship was marginally significant (r^2^ = 0.64, P = 0.06). However, we found a significant and negative correlation between δ^15^N change and soil bulk density (r^2^ = −0.86, P = 0.008).

Assuming that there is no isotopic fractionation during decomposition^25,26^, the isotopic-mixing model indicated that the percentage contribution of C_3_ plants (i.e. woody species) to SOC in the encroached grassland increased from 24.7% in the 0–10 cm soil layer to 31.6% in the 60–100 cm soil layer at the 1500 mm MAP site. From the 900 to the 300 mm MAP sites, the contribution of C_3_ plants to SOC generally decreased with increasing depth in the encroached grasslands (Table 3). The C_3_ contribution to SOC ranged from 64.6 to 24.7% in the uppermost soil layer and between 8.59 to 35.7% in the 60–100 cm soil layer. The lowest C_3_ contribution was at the 750 mm MAP site which ranged from 3.6 to 17.6%. We acknowledge that the isotopic-mixing model used here does not take into account the fractionation of δ^13^C with depth due to decomposition^27^.

**Table 3:** Carbon derived from encroaching C_3_ woody plants in encroached sites.

MAP (mm)	0–10 cm Mean ± SE (%)	10–30 cm Mean ± SE (%)	30–60 cm Mean ± SE (%)	60–100 cm Mean ± SE (%)
1500	24.7 ± 3.4	19.7 ± 5.8	30.8 ± 2.1	31.6 ± 1.4
900	64.6 ± 2.5	29.5 ± 2.74	33.6 ± 2.9	35.7 ± 5.7
750	17.6 ± 4.7	3.6 ± 0.5	6.6 ± 1.5	8.59 ± 0.8
500	47.1 ± 2.6	23.7 ± 7.0	41.2 ± 4.8	16.5 ± 4.2
350	46.6 ± 2.0	20.6 ± 3.4	41.2 ± 4.8	18.5 ± 5.5
300	34.8 ± 6.2	27.0 ± 1.9	26.5 ± 1.69	22.0 ± 4.2

Our study lacked a direct test of SOC change independent of other covarying factors, such as fire, grazing intensity and microbial activities. Nonetheless, our findings agree with several studies^8,28,29^ that found a negative correlation between change in SOC and MAP due to shrub encroachment. Our results, however, were contrary to a review by Barger *et al*.^11^ who showed that C response to shrub encroachment was highly variable and unrelated to MAP. Additionally, our findings also show that shrub encroachment sequesters more C at MAP < 900 mm while the study by Jackson and colleagues^8^ found a threshold value of approximately 400 mm MAP. The variance in the threshold MAP might be due to large differences in SOC level between their studies and our study sites, with their most arid site (230 mm MAP) having more C than our 500 mm MAP site.

The increase of SOC in the drier sites due to shrub encroachment is in accordance with the findings from several studies^8,30,31^. This increase in SOC agrees with the well-described island of fertility^14,28,32^. Shrubs and trees develop extensive root systems that extract nutrients from the horizontal and vertical planes^30^, leading to local nutrient recycling and greater biomass production associated with organic matter accumulation^29,30^. The accumulation of SOC in the drier shrub-encroached grasslands can also be due to greater rates of primary productivity in the more arid sites because the encroached grasslands had greater biomass than open grasslands. Greater primary productivity increases soil organic matter input in the soil beneath deep-rooted shrubs through increased surface litter and accumulation of complex organic compounds, which are resistant to decomposition^14,24^. The humid site (MAP = 1500 mm) had more C in the open grassland compared to the encroached grassland. This finding for our most humid site was contrary to that of Chiti *et al*. (2016)^33^ who found that woody plant encroachment resulted in the increase in SOC. However, our results were consistent with the findings of Jackson *et al*.^8^. In high-precipitation areas, grasslands are highly productive and allocate a large proportion of SOC belowground with greater root turnover rates^8^.

Based on the carbon isotopes, this study also indicated that the current organic matter inputs are not in isotopic equilibrium with soil organic matter in shrub-encroached grasslands. The δ^13^C values of the plant foliage was generally lower than those of soil organic carbon across all sites. In the shrub-encroached grasslands, δ^13^C values of organic inputs were characteristic of C_3_ plants (−32 to −28‰) while δ^13^C values of the associated SOC was −25 to −14‰. These contrasting values suggest that our shrub-encroached grasslands dominated by C_3_ plants were once occupied by C_4_ grasses.

δ^13^C soil values in our open grasslands (−18 to −12‰) where generally comparable to other C_4_ grasslands and savannas across the world (−16 ± 2.2‰)^34^. Thus, the δ^13^C values across all our six study sites showed that SOC in open grasslands came from a C_4_ dominated ecosystem. The discrepancy between δ^13^C values of vegetation and soils in open grasslands of our drier study sites may reflect lateral intrusion of C_3_ plant roots from the shrub-encroached grasslands. Shrubs in semi-arid region have been shown to have extensive roots that can extend to several meters^35^.

In our drier sites (500, 350 and 300 mm MAP sites), a strong memory of C_4_ plants that once dominated these sites was shown by SOC δ^13^C values at depths deeper than 30 cm, while the humid sites (1500 and 900 mm MAP) showed a significant δ^13^C change across the whole range of depth studied. This may indicate a quick SOC turnover in humid sites compared to semi-arid sites. It may also suggest that shrub-encroachment is more recent in semi-arid sites relative to the humid sites. Recent changes in C_4_ to C_3_ productivity might be evident in the δ^13^C of SOC near the surface were SOC turnover is most rapid and current organic input are concentrated.

A particularly strong memory of C_4_ plants that occupied the shrub-encroached grassland was very evident in δ^13^C values for our 750 mm MAP sites, which ranged from −14 to −12‰ across the soil depth. This finding indicates that organic carbon at this shrub-encroached site was exclusively from C_4_ plants and that little SOC was derived from the present C_3_ vegetation. This very strong C_4_ memory can be attributed to the high clay content of the soil at the site^34^. The finding is consistent with other studies which showed slow turnover of clay associated organic carbon^15,34^. Organic carbon associated with clay soils contains aliphatic hydrocarbons which are inert from carbon turnover^36^.

In the 1500 mm MAP site the δ^13^C signature in encroached grasslands indicated that new SOC from woody plants has been incorporated into the soil. However, the finding that this humid site had low SOC may indicate that the rate at which SOC is being lost is higher than that being added by the C_3_ plants. The woody plants have added approximately 24.7% of SOC compared to 50.5% lost due to encroachment. This humid site had also the lowest SOC and SN compared to our other humid sites. The isotopic mixing model also shows that C_3_ SOC contribution for this site increased with depth from 24.3% in the 0–10 cm depth to 31.6% in the 60–100 cm depth. These findings may indicate high nutrient leaching accelerated by the sandy soil type and high MAP.

Although the encroached grasslands are dominated by C_3_ plants, the bulk of the SOC likely comes from the C_4_ plants according to the mixed-isotopic model we used. The higher SOC δ^13^C deeper in the soil may be interpreted as supporting this conclusion, although this depends on there being limited discrimination against ^13^C during respiration/decomposition^25,26^. We have shown elsewhere, using aerial and fixed-point photography, that several of the sites (300 mm, 350 mm and 750 mm MAP) were open grasslands 20 to 50 years ago^31–34^. Although 1500 mm MAP site possibly indicated a decrease in the contribution of the C_4_ plants in SOC with increasing depth, the bulk of the SOC is still potentially derived from the C4 plants. This decrease could also mean that the 1500 mm MAP site has been encroached for a much longer period than in the other sites.

Soil N was higher in the encroached grasslands compared to open grasslands at the 900, 500, 350 and 300 mm MAP sites over the 0–10 cm soil depth probably because most of the encroaching woody species at these sites are leguminous N-fixing plants such as *Vachellia erioloba*, *Vachellia* *karroo* and Senegalia *mellifera*. N_2_-fixing plants ameliorate and increase N in the soil^35^. However, at the 1500 mm MAP site, soil nitrogen was significantly higher in the open compared to the encroached grasslands, which is consistent with the SOC trends at this site. This finding is consistent with results from other African savanna ecosystems where sites encroached by woody species have been compared with open grasslands^37–39^.

There was a general decrease of plant material δ^15^N values with MAP. This finding is consistent with many other studies that showed a negative correlation between δ^15^N and MAP^40–42^. A plausible explanation for this decrease in δ^15^N with increasing MAP is that photosynthetic activity become more water-than nutrient- limited^41^. Arid environments have high nutrient availability therefore have a more open N cycle resulting in a less negative δ^15^N compared to humid ecosystems.

There were no significant differences in δ^15^N values between shrub-encroached and open grasslands across all depths as would be expected if the source of the increase in soil N pools was N fixation^21,37^. This might suggest other processes affecting ^15^N concentration, such as N pumping from deeper soil layers and isotope fractionation during litter decomposition^21,43^ and denitrification^39^, to be contributing to soil nitrogen levels in the shrub-encroached grasslands.

This study generally showed shrub encroachment may result in SOC gains in areas with a threshold value between 750 mm and 900 mm MAP. Most of the SOC in the shrub-encroached grasslands came from the C_4_ plants that previously occupied the area. The significant difference between our study and the North American study of Jackson *et al*.^8^ which showed a threshold of 400 mm indicates that extrapolation across continents is problematic, as has been indicated for savannas elsewhere^44^.

## Methods

Soil samples were collected between October 2013 and March 2014 at six different sites. The site are described in Table 1 according to Mucina and Rutherford (2010)^45^. Each site was divided into encroached and unencroached sections, using adjacent sections to minimize differences in topography. In three of our sites (300, 350 and 750 mm MAP) there is documented evidence^46–49^, while for the other three sites we have strong oral evidence, suggesting that our sites were once open grasslands 20–50 years ago. Encroached sites were defined as those areas with >40% shrub cover. Six 1 m-deep and 1 m-wide pits were dug at random at each site in each section. Within each pit, two soil cores of 100 cm^3^ each were sampled (drilled horizontally into the side of the pit) at centre of each of the four different depth intervals (0–10, 10–30, 30–60 and 60–100 cm). One core was used to quantify soil bulk density (BD) and soil texture, and the other core was used to determine soil organic carbon (SOC), total nitrogen (TN), δ^13^C and δ^15^N values. Bulk density and soil texture were quantified using the core method and pipette method, respectively^50,51^. The soil cores designated for SOC, TN, δ^13^C and δ^15^N were air-dried and passed through a 2 mm sieve. Soil samples for SOC and δ^13^C were treated to remove soil carbonates. To remove soil carbonates, soil samples were treated with 1N H_2_SO_4_/5% FeSO_4_ and double-checked for complete inorganic C removal^51^. Soil samples were air-dried at 40 °C and then sieved (2-mm mesh). Generally, our soil samples were free of rocks. Total SOC and N were measured using a Europar elemental analyser (Germany) at BemLabs (Somerset West, Western Cape). The SOC and N stocks were calculated using the following equation:

1 $${S}_{s}={x}_{1}{x}_{2}{x}_{3}$$

where S_s_ is the SOC or nitrogen stocks (Mg C ha^−1^); x_1_ is the C or N concentration in the soil material (%); x_2_ is the soil bulk density (Mg m^−3^); x_3_ is the thickness of the soil layer (cm).

We collected plant foliage from the five most abundant species at each site in each landcover area (encroached and open grassland). We sampled litter layer composed of plant debris in different stages of decomposition using a 15 cm × 15 cm wooden frame around each station. Both the plant material and the litter layer were air dried for 3–4 days and then oven dried at 60 °C. The plant foliage and the litter-layer material were then ground before the determination of their δ^13^C and δ^15^N signatures.

For δ^13^C and δ^15^N values, c. 40 mg of soil, 2 mg of plant foliage and 5 mg of litter- layer material was weighed into tin capsules (Elemental Microanalysis Ltd., Devon, UK) and combusted in a Thermo Flash EA 1112 series elemental analyser; the gases were fed into a Delta Plus XP isotope ratio mass spectrometer (Thermo Electron Corporation, Milan, Italy). The C isotopic ratio of a sample were expressed relative to the Pee Dee Belemnite standard^52^ and the N isotopic ratios was expressed relative to atmospheric nitrogen^53^.

Assuming complete and unbiased mixing in the soil, we used the δ^13^C values of soils (Fig. 5) and our mean δ^13^C values for the plant material of C_3_ (shrub) and C_4_ (grass) species (Fig. 4) present at our sites to estimate the relative proportion (%) of soil organic matter derived from C_4_ and C_3_ photosynthetic pathway sources with an isotopic-mixing model^8^:

2 $${{\rm{FC}}}_{3}=({{\rm{\delta }}}^{13}{{\rm{C}}}_{{\rm{soil}}}-{{\rm{\delta }}}^{13}{{\rm{C}}}_{{\rm{C4}}})/({{\rm{\delta }}}^{13}{{\rm{C}}}_{{\rm{C3}}}-{{\rm{\delta }}}^{13}{{\rm{C}}}_{{\rm{C}}4})$$

where FC_3_ is the carbon fraction derived from C_3_ sources, δ^13^C_soil_ is the measured δ^13^C of the soil sample, δ^13^C_C4_ is the mean δ^13^C (‰) of C_4_ sources, and δ^13^C_C3_ is the mean δ^13^C (‰) of C_3_ sources^37^. The isotopic mixing model used here does not consider δ^13^C fractionation with depth due to decomposition. Several studies have shown no indication of isotopic fractionation during decomposition^25,26^, while several other studies have shown various ^13^C fractionations due to decomposition^27^.

The data on SOC and N concentration, and SOC stocks, δ^15^N and δ^13^C values attributable to the effects of shrub encroachment and soil depth were analysed using a multivariate ANOVA analysis (MANOVA) to reduce problems with Type I error because we examined several dependent variables simultaneously. The datasets used in this study are available from the corresponding author on request.
